# Heterozygous Missense Variants in the ATPase Phospholipid Transporting 9A Gene, *ATP9A*, Alter Dendritic Spine Maturation and Cause Dominantly Inherited Nonsyndromic Intellectual Disability

**DOI:** 10.1155/humu/7085599

**Published:** 2025-03-05

**Authors:** Amélie Cordovado, Yvan Hérenger, Coline Cormier, Estrella López-Martín, Hannah Stamberger, Laurence Faivre, Anne-Sophie Denommé-Pichon, Antonio Vitobello, Hamza Hadj Abdallah, Giulia Barcia, Thomas Courtin, Beatriz Martínez-Delgado, Eva Bermejo-Sánchez, María J. Barrero, Brooklynn Gasser, Stéphane Bezieau, Sébastien Küry, Sarah Weckhuysen, Frédéric Laumonnier, Annick Toutain, Marie-Laure Vuillaume

**Affiliations:** ^1^Imaging Brain and Neuropsychiatry, iBraiN U1253, INSERM, University of Tours, Tours, France; ^2^Genetica AG, Human Genetics and Genetic Counselling Unit, Zurich, Switzerland; ^3^Genetic Center, Rare Diseases Reference Center On Developmental Anomalies and Malformative Syndromes, FHU TRANSLAD, University Hospital, Dijon, France; ^4^Institute of Rare Disease Research, Instituto de Salud Carlos III (ISCIII), Madrid, Spain; ^5^Applied & Translational Neurogenomics Group, VIB Center for Molecular Neurology, VIB, Antwerp, Belgium; ^6^Translational Neurosciences, Faculty of Medicine and Health Science, University of Antwerp, Antwerp, Belgium; ^7^Department of Neurology, Antwerp University Hospital, Antwerp, Belgium; ^8^INSERM, Unit 1231 GAD Team, Burgundy University, Dijon, France; ^9^Medical Genomics Laboratory, FHU TRANSLAD, University Hospital, Dijon, France; ^10^Rare Diseases Genomic Medicine Department, Necker-Enfants Malades University Hospital, Paris, France; ^11^Medical Affairs, Ambry Genetics, Aliso Viejo, California, USA; ^12^Medical Genetics Department, University Hospital, Nantes, France; ^13^Thorax Institute Research Unit, INSERM, CNRS, Nantes University, Nantes, France; ^14^Genetics Department, University Hospital of Tours, Tours, France

## Abstract

Intellectual disability is a neurodevelopmental disorder, affecting 2%–3% of the population, with a genetic cause in the majority of cases. *ATP9A* (Online Mendelian Inheritance in Man (OMIM)⁣^∗^609126, NM_006045.3) has recently been added to the list of candidate genes involved in this disorder with the identification of biallelic truncating variants in patients with a neurodevelopmental disorder. In this study, we propose a novel mode of inheritance for *ATP9A*-related disorders with the identification of five de novo heterozygous missense variants (p.(Thr393Arg), p.(Glu400Gln), p.(Lys461Glu), p.(Gly552Ala), and p.(His713Asp)), in patients with intellectual disability. In a patient with a similar phenotype, we also identified two truncating variants in *ATP9A* (p.(Arg145⁣^∗^), p.(Glu901⁣^∗^)), adding a novel family to the six already described in the literature with the recessive mode of inheritance. Functional studies were performed to assess the pathogenicity of these variants. Overexpression of four selected missense mutant forms of *Atp9a* in HeLa cells and in primary neuronal cultures led to a loss of mature dendritic spines. In HeLa cells, the endosomal localization of the protein encoded by three of these missense variants was preserved whereas the fourth remained blocked in the endoplasmic reticulum. To mimic the effect on neuronal morphology and spine density of nonsense variants, small hairpin RNAs (shRNAs) were used. They induced a decreased expression of *ATP9A*, affecting the neuronal arborization by decreasing the number of dendrites per neuron. Our results therefore demonstrate the pathogenicity of *ATP9A* heterozygous missense variants and confirm the role of *ATP9A* in neuronal maturation and in brain wiring during development. They strengthen the association of *ATP9A* with neurodevelopmental disorders and demonstrate that a double mode of inheritance should be considered for *ATP9A*-related disorders.

## 1. Introduction

Intellectual disability (ID) is a neurodevelopmental disorder (NDD) affecting 2%–3% of the general population and characterized by significant limitations in intellectual functioning and adaptive behavior. The recent use of next-generation sequencing technologies has made gene identification easier, with more than 1400 ID genes known to date, considering all patterns of inheritance, and almost as many candidate genes for this highly heterogeneous disorder (SysID database) [1]. Recently, *ATP9A* was added to this long list of candidates, as biallelic truncating *ATP9A* variants were identified in six families with ID, five being consanguineous [2–4]. *ATP9A*, mapping to 20q13.2 and containing 28 exons, has a full-length transcript of 3.1 kb (NM_006045.3) and encodes a 118 kDa transmembrane protein of 1047 amino acids belonging to the P4-ATPase family. In humans, the P4-ATPase family includes 14 proteins grouped into three different classes, P4A, P4B, and P4C. ATP9A belongs to the P4B class [5]. P4B ATPases are flippases which catalyse phospholipid transport from the exoplasmic side to the cytoplasmic side of the plasma membrane [6–8]. ATP9A is mostly expressed in the brain and is found in the Golgi apparatus and on the recycling endosomes of the cells [9]. To date, no flippase activity has been described for ATP9A, but it has been linked to the recycling of the transferrin receptor, GLUT1 transporter [10], and of proteins from the Wls family [11] to the plasma membrane and to exosome secretion [12]. Here, we highlight a novel mode of inheritance related to *ATP9A* variants by reporting five de novo heterozygote missense *ATP9A* variants, identified in unrelated patients with NDD. We compared their phenotype with that of the patients with biallelic truncating variants previously reported. We assess the pathogenicity of four of these missense variants by conducting functional studies in HeLa cells and in mouse primary hippocampal neuronal cultures. We showed that overexpression of the variants leads to a decreased dendritic spine maturation. These results confirm a role of these *ATP9A* variants in neural circuit formation, consistent with the phenotype of the patients. In addition, we identified two nonsense variants in a single patient adding a novel family to the six previously described with a recessive mode of inheritance. We analysed the impact of these nonsense variants and confirmed that the loss of *ATP9A* expression affects neuronal arborization.

## 2. Materials and Methods

### 2.1. Inclusion of Patients and Genetic Analyses

Affected individuals included in the present study were enrolled together with their healthy biological parents in different programs or centres investigating the molecular basis of developmental disorders in a research or clinical setting and gathered through the GeneMatcher platform [13]. If the genetic analysis was performed in a research setting, the studies were approved by the respective institutional review boards. Written informed consent was obtained from the patient's legal guardians. Clinical information was obtained by review of medical records and examination of affected individuals. Routine clinical genetic and metabolic screenings performed during initial workup were negative in each case, which allowed further investigation and exome or genome sequencing.

Rare heterozygous variants were selected according to their de novo occurrence if known, their absence in the population database Genome Aggregation Database (gnomAD v2.1.1), and their predicted deleterious effect on protein.

In silico prediction was performed with Sorting Intolerant From Tolerant (SIFT4G) [14], PolyPhen-2 [15], MutationTaster [16], and Combined Annotation Dependent Depletion (CADD) [17]. Interpretation of the variants was done according to the recommendations of the American College of Medical Genetics and Genomics (ACMG) [18]. The variants described in this study were submitted to Leiden Open Variation Database (LOVD) (https://www.lovd.nl/).

### 2.2. *ATP9A* Expression Plasmids and Site-Directed Mutagenesis

Experiments were performed using a pcDNA3 expression plasmid containing the full-length *ATP9A* human cDNA sequence fused to an HA tag generously provided by Hye-Won Shin (University of Kyoto). The missense variants p.(Trp393Arg), p.(Glu400Gln), p.(Lys461Glu), and p.(Gly552Ala) were generated by site-directed mutagenesis with the Q5 Site-Directed Mutagenesis Kit (New England Biolabs). The oligonucleotides used to generate these missense mutations were designed using the NEBaseChanger site (https://nebasechanger.neb.com) and included the sequences presented in Table S1. The sequences of all constructs were confirmed by automated DNA Sanger sequencing.

### 2.3. Cell Cultures

HEK293T and HeLa cell lines (American Type Culture Collection (ATCC), CRL-3216 and CCl-2) were cultured and maintained with Dulbecco's modified eagle medium (DMEM) (Gibco, ref 31966047) and 10% of fetal bovine serum (FBS; Eurobio, CVFSVF06-01). Neuro-2a (N2A) cell lines (ATCC, CCL-131) were cultured with Eagle's minimum essential medium (EMEM) (ATCC, 30-2003) and 10% of FBS (Eurobio CVSVF06-01).

For primary neuronal cultures, all mouse experiments were performed according to protocols approved by the University of Tours and Institut National de la Santé Et de la Recherche Médicale (INSERM). Hippocampi were dissected from embryonic Day 17 C57BL/6J mouse embryos (Janvier-Labs). The primary neuronal cultures were prepared as previously described [19]. Dissociated cells were then plated onto glass coverslips coated in poly-D-lysine (Merk) and laminin (Cat# 23017-015, Invitrogen) at a density of 210 cells per square millimeter. The cultures were kept in Neurobasal/B-27, and half of the medium was changed twice a week.

### 2.4. Reverse Transcription Polymerase Chain Reaction (RT-PCR) and Western Blot Analyses in HEK293T Cells

Expression plasmids (wild-type (WT) and mutated forms of *ATP9A*) were transfected into HEK293T cells using Lipofectamine 2000 (Invitrogen). RNA was extracted 48 h after transfection using the Direct-Zol RNA Miniprep Plus Commercial Kit (Zymo Research). Complementary DNA was obtained from 100 ng of mRNA using the sensiFAST cDNA Synthesis Kit (Meridian Bioscience). Primers were designed with Primer3 software (sequences available upon request).

Proteins were extracted using radioimmunoprecipitation assay (RIPA) buffer from Invitrogen and submitted to sonication (4 × 15 c) on ice. Protein samples were then centrifuged, and the pellet was denatured into a buffer containing 8 M of urea, 2% of sodium dodecyl sulfate (SDS), 100 mM of dithiothreitol (DTT), and 375 mM of Tris at pH 6,8 and heated at 37°C for 2 h. Protein samples were processed for sodium dodecyl sulphate–polyacrylamide gel electrophoresis (SDS-PAGE) at 190 V during 3 min. Equal deposition of the proteins was checked by stain-free imaging (Bio-Rad). SDS-PAGE gels were blotted on nitrocellulose membrane using Trans-Blot Turbo (Bio-Rad) for 7 min (1.3 A, 25 V). The membrane was blocked for 1 h with 5% milk diluted in Tris-buffered saline (TBS)-Tween (1% Tween 20). The membrane was incubated overnight in primary antibody for HA tag (1/500 Cat# 11867423001, Merck) diluted in 5% milk in TBS-Tween buffer. After three 10-min washes with TBS-Tween, the membrane was incubated with a secondary goat horseradish peroxidase (HRP)–coupled anti-rat antibody (1/10,000 Cat# 629520, Invitrogen) diluted in 5% milk in TBS-Tween buffer for 1 h at room temperature. Chemiluminescence detection was done using Clarity Western ECL Substrate Kit (Cat# 1705060, Bio-Rad), and the membrane was visualized in a ChemiDoc Touch Imaging System (Bio-Rad). Quantification of protein expression was done using Fiji software (Wayne Rasband, Bethesda NIH), based on three independent experiments, and each band was normalized by the stain-free image. A Kruskal–Wallis test was performed on GraphPad Prism 8.0 software (La Jolla, California, United States) to compare the expression of the different variants.

### 2.5. Immunocytochemistry and Image Analysis in HeLa Cells

Expression plasmids (WT and mutated forms of *ATP9A*) were transfected into HeLa cells using Lipofectamine 2000 (Invitrogen), and HeLa cells were fixed 48 h later with a solution containing 4% paraformaldehyde/4% sucrose in phosphate-buffered saline (PBS). Cultures were then incubated with a blocking buffer (10% donkey serum/0.2% Triton X-100 in PBS) for 1 h, washed with PBS, and incubated for 1 h with the following primary antibodies diluted in 3% donkey serum/0.2% Triton X-100 in PBS buffer: monoclonal rat anti-HA antibody (1/200 Cat# 11867423001, Merck) and mouse anti-calnexin (1/500, Cat# MA3-027, ThermoFisher Scientific) to target endoplasmic reticulum and rabbit anti–early endosome antigen 1 (EEA1) (1/500, Cat# PA1-063A, Invitrogen) to target endosomes. After several washes in PBS, HeLa cells were incubated for 45 min with the following secondary antibodies diluted in 3% donkey serum/0.2% Triton X-100 in PBS buffer: FluoProbes 488 goat anti-rat antibody (1/500, Cat# A-11006, ThermoFisher), FluoProbes 594 donkey anti-rabbit antibody (1/500, Cat#FP-SD5110, Interchim), and FluoProbes 594 donkey anti-mouse antibody (1/500, Cat# A21203, ThermoFisher). After three washes in PBS, the fixed and stained HeLa cells were mounted in ProLong Diamond Antifade Reagent (Cat# P36391, Invitrogen). Sequential acquisitions were made, and high-resolution *z* stack images of cells were taken with the × 63 objective of a laser-scanning confocal microscope SP-8 (Leica) with an optical section separation (*z* interval) of 0.3 *μ*m. Images were generated by the in-built Leica Application Suite X (Leica). Median projections were made for image analysis. Colocalization quantification was performed using Just Another Colocalization Plugin (JACoP) of Fiji software (Wayne Rasband, Bethesda NIH) to obtain Pearson's and Mander's coefficients. Statistical analysis was done with a Kruskal–Wallis test on GraphPad Prism 8.0 software (La Jolla, California, United States).

### 2.6. Immunocytochemistry and Image Analysis in Primary Neuronal Culture

After transfection with *ATP9A* expression plasmids (WT or mutants), neurons were fixed at 6 days in vitro (DIV) for colocalization analyses and neuronal arborisation measurements or at DIV 13 for spine density assessment with a solution containing 4% paraformaldehyde/4% sucrose in PBS. After incubation with blocking buffer, fixed neurons were incubated with the monoclonal rat anti-HA antibody (1/200 Cat# 11867423001, Merck) and the mouse anti-PSD95 antibody (1/200, Cat#MA1-046, ThermoFisher) diluted in 3% Donkey serum/0.2% Triton X-100 in PBS buffer. After the washes, they were incubated with the secondary antibody FluoProbes 594 goat anti-rat (1/500, Cat#A110007, Life Technologies) and FluoProbes 680 goat anti-mouse (1/500, Cat#A21057, ThermoFisher) diluted in 3% Donkey serum/0.2% Triton X-100 in PBS buffer. After several washes in PBS, the fixed and stained neurons were mounted in ProLong Diamond Antifade reagent (Cat# P36391, Invitrogen). Sequential acquisitions were made, and high-resolution *z* stack images of neurons were taken with the × 63 objective of a laser-scanning confocal microscope SP-8 (Leica) with an optical section separation (*z* interval) of 0.3 *μ*m. ATP9A transfection of every neuron has been checked before image generation. Images were generated by the in-built Leica Application Suite X (Leica). Maximal projections were made for image production, EGFP labelling was used as a tracer of neuronal and spine morphology and PSD95 as a marker of postsynaptic density. The spine density measurements were performed using the Fiji software (Wayne Rasband, Bethesda NIH). Mature spines and immature spines were counted based on their head diameter. Mature spines presented a head diameter of more than 0.6 *μ*m. Immature spines presented a head diameter of less than 0.6 *μ*m. Three segments from different dendrites were analysed for each cell. Quantification was based on three independent experiments with more than 16 cells of each type analysed. Mann–Whitney statistical test was used to compare total spine number and to evaluate variations of spine maturity (GraphPad Prism 6.0 software, La Jolla, California, United States). To measure neuronal arborisation, EGFP was used as a tracer of neuronal morphology. Images were analysed with the filament tracer plugin of the IMARIS software (Oxford Instruments). Quantification was based on three independent experiments with more than 20 neurons of each type that were analysed. Kruskal–Wallis statistical test was used to compare total neurite length and total neurite number and to assess arborisation complexity (GraphPad Prism 6.0 software, La Jolla, California, United States).

### 2.7. Knockdown Experiments

Plasmids containing a small hairpin RNA (shRNA) targeting *ATP9A* mouse transcripts as well as plasmids containing a scrambled shRNA were used (Cat# MSH031243-CU6-a (OS613088) GeneCopoeia). The expression of the shRNA was driven by a U6 promoter. These constructs contained the puromycin resistance gene and an EGFP gene which expresses EGFP constitutively in mammalian cells. Both effective shRNA and noneffective shRNA were first transfected in N2A cells to assess their efficiency to repress the expression of endogenous *ATP9A*. shRNAs were transfected (Lipofectamine 2000, Invitrogen) in N2A cells 24 h after seeding, and RNA extraction was performed 48 h after transfection using the Direct-Zol RNA Miniprep Plus Commercial Kit (Zymo Research). Complementary DNA was obtained from 400 ng of mRNA using the PrimeScript RT Reagent Kit (Perfect Real Time) (Cat# RR037A, Takara). Quantitative polymerase chain reaction (qPCR) experiments were run in duplicates with 500 ng of cDNA on the LightCycler 480 (Roche Diagnostics, Meylan, France) using the SYBR Green Master Mix commercial kit (Applied Biosystem, Foster City, California, United States). Relative expression was assessed using the advanced E-method from LightCycler software (Roche Diagnostics, Meylan, France). Hypoxanthine phosphoribosyltransferase (hprt), glyceraldehyde-3-phosphate dehydrogenase (gapdh), and beta-actin were used as reference genes for normalization. To measure the expression levels of *ATP9A* in each condition, reverse transcription-quantitative polymerase chain reaction (RT-qPCR) analyses were performed on three independent transfections.

Neurons were transfected (Lipofectamine 2000, Invitrogen) at DIV 10 with noneffective or effective shRNA. They were fixed at DIV 13, and spine density and arborisation measurements were performed as described previously [20]. The EGFP was used to assess neuronal morphology. Quantification was based on three independent experiments leading to more than 17 cells analysed for each condition.

## 3. Results

### 3.1. Description of *ATP9A* Variants

**Figure 1:**
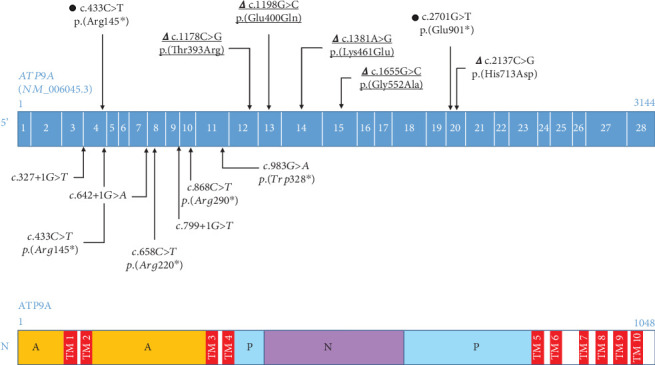
Distribution of *ATP9A* heterozygous variants. The genomic structure of *ATP9A* gene including 28 exons (blue boxes) is represented. cDNA numbering above the *ATP9A* gene is according to NM_006045.3. The ATP9A protein structure is represented below the gene. Amino acid numbers are indicated above the protein. Variants are displayed as changes at DNA (c.) and protein levels (p.). Triangles and black circles indicate missense and truncating variants (frameshift or nonsense), respectively. Biallelic truncating variants already described in the literature [2–4] are indicated in italics below the gene. Variants for which functional studies have been conducted are underlined. A: actuator domain, N: nucleotide binding domain, P: phosphorylation domain, TM: transmembrane domain.

In the present study, we report seven *ATP9A* variants including five de novo missense variants (Figure S1), and two nonsense variants identified in six patients with ID. A schematic view of *ATP9A* gene and the distribution of all variants are presented in Figure 1. All missense variants were absent in the control population database gnomAD (v4.0.0). Two missense variants, p.(Thr393Arg) and p.(His713Asp), were located in Exon 12 and in Exon 20 in the phosphorylation domain whereas the other missense variants, p.(Glu400Gln), p.(Lys461Glu), and p.(Gly552Ala), were located, respectively, in Exons 13, 14, and 15 corresponding to the nucleotide binding domain of ATP9A. The variant p.(Thr393Arg) affects a highly conserved motif among P-type ATPases, the DKTGT motif involved in phosphorylation during the reaction cycle of ATPases. All these five variants were predicted to be pathogenic by most of in silico prediction programs (Table S2). To assess the hypothesis of a gain of function or dominant negative effect, four of these variants were selected for functional studies and were overexpressed in HEK293T cells and in primary hippocampal neurons from mouse embryos.

Two rare nonsense variants, p.(Arg145⁣^∗^) and p.(Glu901⁣^∗^), located, respectively, in Exon 4 and Exon 25, were identified in a sixth unrelated patient. The variant p.(Arg145⁣^∗^), already reported by Meng et al., occurred de novo whereas the variant p.(Glu901⁣^∗^) was inherited from the unaffected mother. Due to their location, these nonsense variants are very likely to induce nonsense-mediated mRNA decay (NMD) and to lead to a loss of function. In order to model the impact of a loss of function effect triggered by *ATP9A* truncating variants, notably biallelic variants, we performed knockdown experiments using shRNA in primary hippocampal neurons from mouse embryos.

### 3.2. Clinical Description (Table 1 and Supporting Data)

Detailed clinical information was available for five individuals. The information provided for the individual with the p.(Lys461Glu) was limited. This patient was tested for ID associated with hypotonia, poor balance, microcephaly, poor growth, and a few nonspecific dysmorphic features.

The other five individuals, two males and three females, all isolated cases, were aged 2 1/2, 10, 10, 14, and 44 years at the time of the reporting. Four of them were born to unrelated Caucasian parents, and the last one was born to consanguineous Moroccan parents.

The reason for referral of the four individuals with missense variants was global or motor delay for two of them and ID and epilepsy for the two others. All were born at term or near term, with normal Apgar scores, following unremarkable pregnancy and delivery. Birth parameters were in the normal range for the three youngest individuals who had a normal height on last examination (parameters unknown for the oldest). Two individuals had overweight, possibly due to medication in one of them. One patient had marked feeding difficulties with gastro-oesophageal reflux, vomiting, and food aversion which required gastrostomy. None of the patients had visceral or skeletal malformations but two had orthopedic problems (scoliosis in one and joint retractions in two) as a consequence of their neurological condition, and one experienced several bone fractures during seizures. Minor and nonspecific dysmorphic facial features were reported in two patients, but no recognizable phenotype was observed.

Delayed development was reported for all individuals. Motor skills were known for three patients and were delayed (one being unable to walk independently at 14 years), and two, among three for whom information was available, had hypotonia in infancy and early childhood and fine motor problems. Language was also impaired in all individuals: one had language delay but the ability do to short sentences, one was unable to do sentences at 2 1/2 years, and two had no language at 10 and 14 years. Apart from the youngest patient who is only 2 1/2 years old, all individuals were considered having severe ID and received special education. Three patients had autistic-like features, either a ritualized daily life with a rigid nature in one or stereotypic movements, such as flapping hands, in the two others, one of them also having poor contact and hetero- and autoaggressive behavior. These latter two patients developed progressive spasticity with brisk tendon reflexes and joint retractions, resulting in the loss of the ability to walk for one of them who was treated with botulinum toxin injections. These two patients also had strabismus with significant astigmatism, nystagmus in one of them, and amblyopia in the other, and visual evoked potentials were reported as immature in both of them. Auditory evoked potentials suggested left auditory neuropathy in one of these individuals.

Three individuals suffered from epilepsy (but the fourth was only 2 1/2 years old). One had generalized tonic–clonic seizures since the age of 11 years, requiring two antiepileptic drugs. The oldest patient had severe refractory epilepsy starting at 1 year, diagnosed as Lennox–Gastaut syndrome, and still requiring five medications despite anterior callosotomy performed at 18 years. Only one individual had a mild microcephaly at −3 SD.

Variable brain MRI abnormalities were reported in three patients and consisted in cortical atrophy (2 patients), partial agenesis of the corpus callosum (1), delayed myelination (1), epiphyseal and pars intermedia cysts (1), and nonspecific flair hypersignal of the posterior parietal periventricular white matter (1). The MRI of the oldest individual, performed at the age of 35 years, showed sequela of her callosotomy and a mild asymmetry of hippocampi.

By comparison, the phenotype of the individual with the two nonsense variants was very similar. She was referred for global delay. She had hypotonia and gross and fine motor problems, being unable to walk without support at the age of 10 years. Her language was limited to a few words. She had severe ID and needed special education. She was described as having anxiety and abnormal social interactions and behavior. She had partial seizures consisting in tonic upward gaze and disconnection with the environment since the age of 7 1/2 years. She had erratic eye movements due to a cortical visual defect. She also had marked feeding difficulties with gastro-oesophageal reflux, vomiting, and dysphagia. Like the individuals with missense variants, she had no congenital malformation or dysmorphic features. The only difference observed with the other patients was failure to thrive. This patient was born at term and small for the gestational age and later still had growth retardation with a height at −2.67 SD and a weight at −2 SD.

### 3.3. Expression and Subcellular Localization of WT and ATP9A Mutants

**Figure 2:**
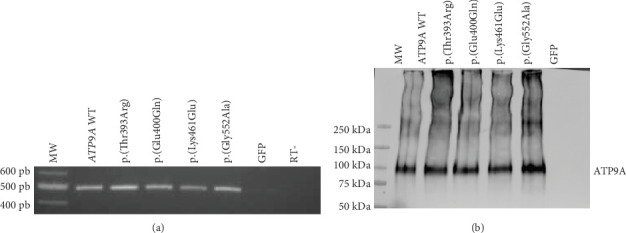
*ATP9A* expression at the RNA level (a) or at the protein level (b) in HEK293T cells. Expected size of the PCR product is 494 bp. The expected molecular weight of ATP9A main isoform is 120 kDa. Three independent experiments were performed, and one representative experiment is shown (bp: base pairs, kDa: kilodalton, WT: wild type, RT-: no reverse transcriptase control, GFP: green fluorescent protein, MW: molecular weight).

**Figure 3:**
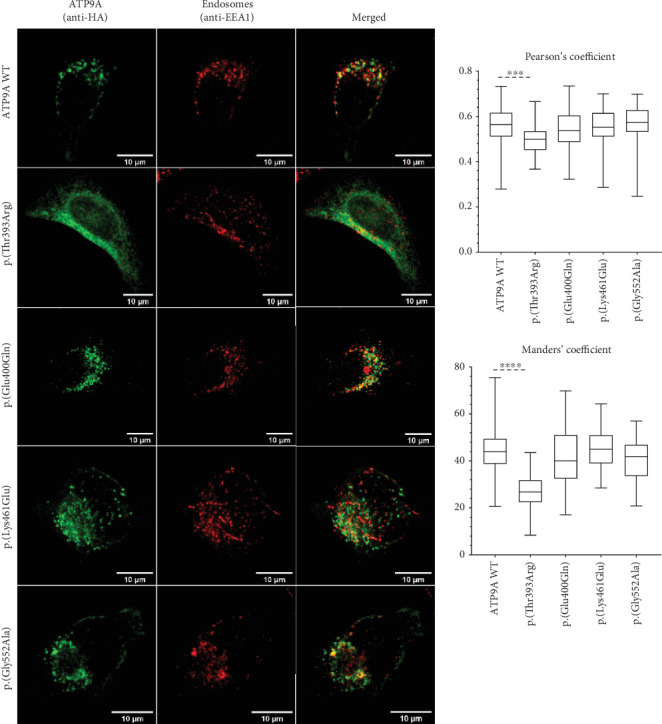
Colocalization of wild-type or mutants forms of ATP9A protein overexpressed in HeLa cells with EEA1. ATP9A is tagged in green by using an anti-HA antibody. Endosomes are tagged in red using an anti-EEA1 antibody. Colocalization was quantified on median projections using JaCoP plugin from ImageJ software based on Pearson's correlation coefficient and Mander's coefficient. Data shown are median, first and third quartiles, and minimal and maximal values. More than 38 cells from three independent experiments were analysed. Statistical analysis was performed with GraphPad Prism 8.0 software (La Jolla, California, United States) by using a Kruskal–Wallis test; ⁣^∗∗∗^*p* < 0.001 and ⁣^∗∗∗∗^*p* < 0.0001.

**Figure 4:**
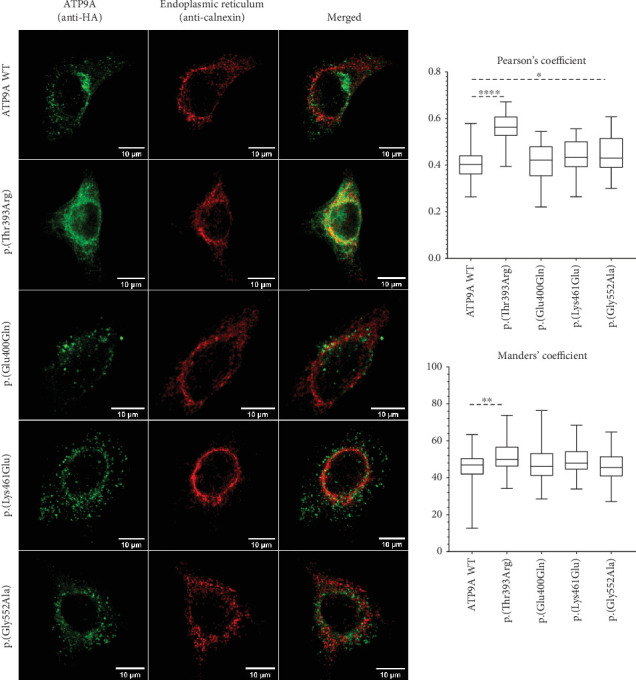
Colocalization with calnexin of wild-type or mutants forms of ATP9A protein overexpressed in HEK293T cells. ATP9A is tagged in green by using an anti-HA antibody. Endoplasmic reticulum is tagged in red using an anti-calnexin antibody. Colocalization was quantified on median projections using JaCoP plugin from ImageJ software based on Pearson's correlation coefficient and Mander's coefficient. Data shown are median, first and third quartiles, and minimal and maximal values. More than 38 cells from three independent experiments were analysed. Statistical analysis was performed with GraphPad Prism 8.0 software (La Jolla, California, United States) by using a Kruskal–Wallis test; ⁣^∗^*p* < 0.05, ⁣^∗∗^*p* < 0.01, and ⁣^∗∗∗∗^*p* < 0.0001.

WT and mutant forms of *ATP9A* were overexpressed first in HEK293T cells in order to assess whether these variants could disturb *ATP9A* expression, stability, and subcellular localization. WT and mutant forms of *ATP9A* were well expressed at the RNA level (Figure 2(a)) and at the protein level (Figure 2(b)). Proteins seemed to be equally expressed (*p* = 0.8268) in HEK293T cells (Figure 2(b)), suggesting that the mutant proteins are produced and stable. As expected for an endosomal protein, the WT protein formed punctate dots that colocalize with EEA1, a marker of recycling endosomes (Figure 3). The mutant forms, p.(Glu400Gln), p.(Lys461Glu), and p.(Gly552Ala), presented the same localization, whereas the variant p.(Thr393Arg) was not efficiently localized to the endosomes but seemed to colocalize with calnexin, a marker of the endoplasmic reticulum (Figure 4). The modification of the highly conserved DKTGT motif therefore seems to block the protein into the endoplasmic reticulum. A slight difference in colocalization with the endoplasmic reticulum was also found for the variant p.(Gly552Ala) only when quantified with Pearson's coefficient. No difference was observed between the WT form and this mutant form regarding colocalization with endosomes.

The WT and mutant constructs of *ATP9A* were next overexpressed in primary hippocampal neuronal cultures. ATP9A was located, whatever the condition (overexpression of the WT form or of the mutant forms of ATP9A), in the three main compartments of the neurons (dendrites, axon, and soma). Unlike the three others which formed punctate dots, the mutant p.(Thr393Arg) exhibited a more diffuse localization in the neurons (Figure S2).

### 3.4. Spine Maturation Is Reduced With Overexpression of *ATP9A* Variants

**Figure 5:**
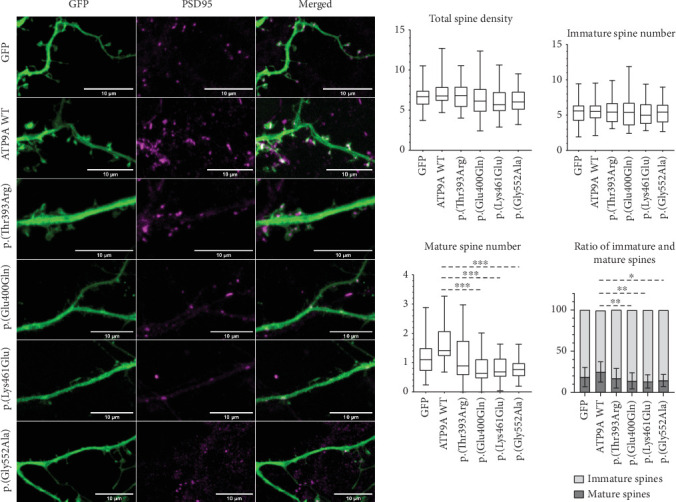
Altered spine density of primary hippocampal neurons after overexpression of *ATP9A* variants. The EGFP was used to show spine morphology (× 63 objective, scale bar: 10 *μ*m). PSD95 in magenta is used to show postsynaptic density of dendritic spines. ATP9A transfection of every neuron has been checked before image generation. To perform the quantification of spine number per 10 *μ*m of dendrite for each condition, mature and immature spines were differentiated according to their head diameter (> 0.6 *μ*m for mature spines and < 0.6 *μ*m for immature spines). Three independent experiments were conducted leading to the analysis of more than 19 neurons for each condition. Means ± SD are represented on the graph presenting the ratio of mature and immature spines. Median, first and third quartiles, and minimal and maximal values are represented on the other graphs. Statistical analysis was done by using a Kruskal–Wallis test with GraphPad Prism 8.0 software, La Jolla, California, United States: ⁣^∗^*p* < 0.05, ⁣^∗∗^*p* < 0.01, and ⁣^∗∗∗^*p* < 0.001.

The impact of the WT and mutant forms of *ATP9A* on neuronal development and morphology was assessed by measuring the branching complexity and the spine density. Overexpression of the different variants neither affected neuronal morphology, dendritic growth, branching complexity (data not shown), nor spine density but had an impact on spine maturation. The variants p.(Glu400Gln), p.(Lys461Glu), and p.(Gly552Ala) led to a decrease in the number of mature spines and to a change in the percentage of mature and immature spines (Figure 5) while the variant p.(Thr393Arg) did not affect the total spine number or the mature spine number. It is likely that this mutant protein cannot reach the endosomes and consequently cannot exert a deleterious effect on spine maturation. Taken together, these findings suggest that ATP9A plays an important role in the maturation of dendritic spines. This role seems to be altered when ATP9A is mutated but still present in the endosomes.

### 3.5. Neuronal Morphology, Dendritic Growth, and Branching Complexity Are Reduced After Downregulation of *ATP9A* Expression

**Figure 6:**
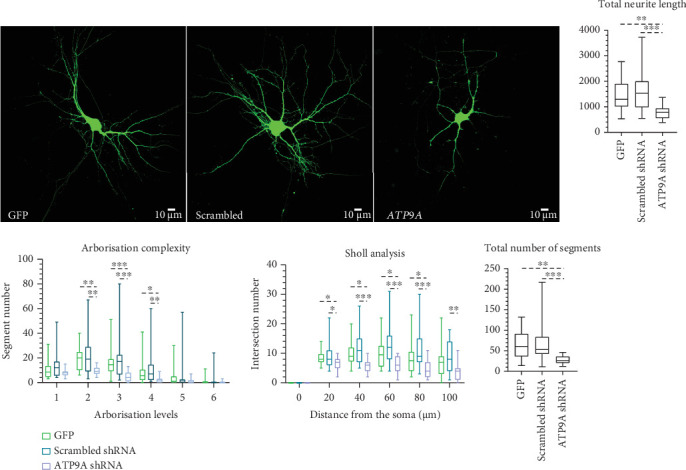
Altered arborisation complexity of primary hippocampal neurons after inhibition of *ATP9A* expression. The EGFP was used to show neuronal morphology (× 63 objective, scale bar: 10 *μ*m). Quantification of arborisation levels, number of neurites, total neurite length, and Sholl analysis were performed using the filament tracer software of IMARIS. Three independent experiments have been done leading to the analysis of more than 19 neurons for each condition. Means ± SD are represented on the graphs. Statistical analysis was done by using a Kruskal–Wallis test with GraphPad Prism 8.0 software (La Jolla, California, United States): ⁣^∗^*p* < 0.05, ⁣^∗∗^*p* < 0.01, and ⁣^∗∗∗^*p* < 0.001.

We modelled the loss of function effect of the nonsense variants by using shRNA transfection to knock down endogenous *Atp9a* expression. Transfection of the effective shRNA in N2A cells resulted in a 70% reduction of *Atp9a* mRNA expression level (data not shown). Effective shRNAs were then transfected in neuronal primary cultures to assess the impact of decreased *Atp9a* expression on neuronal morphology and spine density. We found that the decreased expression of *Atp9a* had neither impact on spine density nor on spine maturation but led to a decrease in arborisation complexity starting at Level 2 with a decrease of the number of neurites and of the total neurite length. (Figure 6).

## 4. Discussion

Recently, seven truncating variants (four nonsense and three splice site variants) were reported in the *ATP9A* gene in nine individuals with NDD, belonging to six unrelated families of various origins (from Asia or Middle East). Five of these variants were at a homozygote state in consanguineous families and two were at a compound heterozygote state in a nonconsanguineous family [2–4]. We report an additional patient with biallelic nonsense variants, one of them being novel, in keeping with the previously described recessive mode of inheritance associated with *ATP9A* loss of function variants. In addition, we show for the first time that *ATP9A* variants may also be associated with a dominant inheritance with the identification of five de novo heterozygous missense *ATP9A* variants in patients with NDD. A dual mode of inheritance has already been reported for an expanding number of genes involved in NDD [21–23], and the pathogenic mechanism could differ according to the type of the mutation. Comparison of the phenotype of the patients from our study and those from the literature, that is, those with heterozygote missense variants and those with biallelic loss of function variants, shows that they are very similar (Table 1). The nine patients from the literature were all reported as having global delay of development with notable speech and fine motor impairment, and six had hypotonia. They all had ID and behavioral problems such as attention deficit hyperactivity disorder or autistic features. Two patients had orthopedic complications which are common in patients with severe ID. Three patients had strabismus which is also common in neurological conditions. Brain MRI showed minor and nonspecific abnormalities in four of the six patients who underwent this investigation. Apart from one individual who had atrial septal defect, which is a very common malformation, none of the patients had visceral or skeletal malformations or striking and recognizable dysmorphic features. Three patients had feeding difficulties including a severe gastro-oesophageal reflux requiring gastrostomy in one of them. Finally, the differences between the two groups of patients which could be highlighted are mild to moderate microcephaly which is more frequent, reported in five individuals from the literature, and failure to thrive affecting six patients from the literature (who had a weight below −2 SD or a BMI below the first percentile, whereas three patients had a small stature), as well as the patient from our study with biallelic nonsense variants. However, the number of patients described until now is too small to allow definite conclusions and further descriptions will be necessary in order to determine whether the clinical picture is more severe with biallelic truncating variants than with heterozygote variants. On the other hand, only one patient from the literature suffered from epilepsy since the age of 3 years and was treated with sodium valproate only.

Functional studies were performed to assess the pathogenicity of four de novo heterozygous missense variants. Each variant was well expressed in HEK293T cells, and in HeLa cells and three of them, p.(Glu400Gln), p.(Lys461Glu), and p.(Gly552Ala) presented the same localization as the WT by forming punctate dots which correspond to recycling endosomes. The variant p.(Thr393Arg) showed however a different localization and seemed to be retained in the endoplasmic reticulum. Interestingly, this variant is located within the highly conserved DKTGT motif, two amino acids downstream to the critical aspartic acid residue that undergoes transient phosphorylation during the catalytic cycle of the P-type ATPases. This catalytic cycle involves four major conformations: E1, E1P, E2P, and E2, and is essential for the role of P4-ATPase in the transport of phospholipids. In human P4-ATPases, ATP11A, ATP11B, ATP11C, and ATP10, the phosphorylation of this residue is inhibited when the aspartic acid is replaced by an asparagine. This inhibition blocks the transition from E1 to E1P conformation and prevents these ATPases from being located to the endosomes [24]. In our study, the variant p.(Thr393Arg), because of its close vicinity to the critical aspartic acid residue, might inhibit the phosphorylation of ATP9A and thus exert a deleterious effect by blocking ATP9A into the endoplasmic reticulum preventing it from fulfilling its function. However, overexpression of this variant did not affect spine maturation, perhaps because this mutant form could not reach the spine. On the contrary, the three other heterozygous de novo missense variants, p.(Glu400Gln), p.(Lys461Glu), and p.(Gly552Ala), did not impact ATP9A subcellular localization but were responsible for a loss of mature spines in primary hippocampal neurons. A link between ATP9A and dendritic spines was recently shown in the study of Meng et al., as they showed that absence of *Atp9a* led to the reduction of secondary dendritic spines in pyramidal neurons of the motor cortex in *Atp9a* knockout mice [3]. Moreover, P4-ATPases enable the recruitment to the plasma membrane of proteins which could play a crucial role in synaptogenesis. As an example, accumulation of phosphatidylserine (PS) induced by P4-ATPAses allows the recruitment of the GTPase CDC42 in the cytosolic plasma membrane in *Saccharomyces cerevisiae*. This protein orchestrates the regulation of actin polymerisation and depolymerisation by interacting with organisers of the actin cytoskeleton [25]. Reorganisation of the actin cytoskeleton results in morphological changes of dendritic spines required for synaptogenesis and for long-term potentiation (LTP) [26]. Furthermore, numerous proteins playing a role in spine maturation or LTP, such as AMPA receptors, are recruited to the postsynaptic density (PSD) through recycling endosomes [27]. By impairing the phospholipid distribution inside the plasma membrane or by disturbing the function of recycling endosomes, ATP9A variations could thus prevent the recruitment of proteins essential for synaptogenesis. Finally, our overexpression studies in primary hippocampal neurons validate the pathogenicity of the four missense *ATP9A* variants tested showing that the first missense variant p.(Thr393Arg) impairs ATP9A localization maybe because it cannot reach the endosome whereas the three others alter neuronal morphogenesis.

We also studied the impact of biallelic loss of function *ATP9A* variants on neuronal development by decreasing endogenous *ATP9A* expression in neuronal cells with the use of shRNAs. Although *ATP9A* expression could not be accurately quantified in neuronal cells, the decrease of *ATP9A* expression observed in N2A cells after shRNA transfection, near to 70%, led us to consider that the knockdown of *ATP9A* could be almost complete in neurons and could reflect the impact of biallelic truncating variants. Interestingly, ATP9A knockdown does not lead to the same effects on neuronal parameters than overexpression of *ATP9A* missense variants. Whereas ATP9A haploinsufficiency did not have any impact on spine maturation, overexpression of *ATP9A* missense variants leads to a loss of mature spines in primary hippocampal neurons showing that overexpression of missense variants has a more deleterious effect than ATP9A loss of function on this specific neuronal parameter. On the contrary, *ATP9A* knockdown impaired neuronal morphology with a less complex arborisation and a decrease of the total number and of the total length of the neurites. These results are in line with the results of Meng et al., as they also observed morphological changes in hippocampal neurons in the absence of *Atp9a* in mice. In their recent study, the intersection numbers of dendritic arborization in the hippocampus of *Atp9a* knockout mice were robustly reduced compared to the WT mice. They also showed that *Atp9a* knockdown by siRNA in rat primary hippocampal neurons led to severe neurite damage and significantly reduced the neurite length [3]. Axon growth has already been linked to other P4-ATPases. A decreased expression of *ATP8A2* in PC12 cells leads to a reduced neurite growth [28], and mutations identified in the mouse are linked to axon degeneration, probably due to a PS exposure on the membrane of the neurites [29]. Our functional studies, by showing a deleterious impact of a decreased *ATP9A* expression on neuronal morphology, confirm a causative role of *ATP9A* bi-allelic truncating variants in NDD.

## 5. Conclusion

To conclude, our results confirm a role of *ATP9A* in neuronal development and demonstrate that a double mode of inheritance should be considered for *ATP9A*-related disorders. Whereas missense variants seem to be associated with a dominant inheritance, truncating variants are associated with a recessive inheritance supporting the hypothesis of two distinct mechanisms of action according to the type of *ATP9A* variant. Pending further clinical descriptions, we also conclude that the phenotype observed in the affected individuals is very similar whatever the mode of inheritance.

## Figures and Tables

**Table 1:** Clinical features observed in the patients carrying a missense ATP9A variant, compared to those of the patients with biallelic nonsense variants.

	**Patients with missense variants**				**Patient with nonsense variants**	
	**Patient 1**	**Patient 2**	**Patient 3**	**Patient 4**	**Patient 5**	**Patients from literature**
*General information*						
Origin	Caucasian	Caucasian	North Africa	Caucasian	Caucasian	Syrian, Turkish, Pakistani, Iranian, Chinese
Gender (age at last exam)	M (14 years)	F (44 years)	F (10 years)	M (2.5 years)	F (10 years)	4 F and 5 M (4–12 years)
Consanguinity (Y/N)	N	N	Y	N	N	Y (5 families)/N (1 family)
cDNA variants	c.1178C>G	c.1198G>C	c.1655G>C	c.2137C>G	c.433C>T & c.2701G>T	3 splice sites variants, 4 nonsense
Protein variant	p.(Thr393Arg)	p.(Glu400Gln)	p.(Gly552Ala)	p.(His713Asp)	p.(Arg145⁣^∗^) and p.(Glu901⁣^∗^)	
Motif of referral/age	Global delay/10 months	Refractory epilepsy, ID	ID, epilepsy/11 months	Motor delay	Global delay	

*Medical information*						
IUGR	N	NA	N	N	Y	2: N; 7: NA
Postnatal statural growth	Normal range	NA	NA	Normal range	Growth failure (−2,67 SD)	3/7
Weight (kg)/SD	Normal range	Overweight	Normal range	Overweight	Growth failure (−2.05 SD)	6/7
Microcephaly	Y (−3 SD)	NA	N	N	NA	5/9
Digestive problems	Gastro-oesophageal reflux, food aversion/gastrostomy	N	Mild constipation	N	Gastroesophageal reflux, dysphagia, episodic vomiting, and chronic diarrhea	3/9 (emesis, gastritis, gastroesophageal reflux)
Orthopedic problems	Scoliosis (arthrodesis) joint retractions	Several fractures due to seizures	Joint retractions (botulinum toxin injections, tenotomies)	N	N	1: Mild scoliosis, pes cavus 1: Joint retractions
Malformations/dysmorphic features	Almond-shaped palpebral fissures, large ears	N	Anteverted nares, R preauricular fistula	Sacral dimple	N	1: Atrial septal defect

*Sensory problems*						
Hearing loss	N	N	Probable L auditory neuropathy	N	N	N
Visual problems	Convergent strabismus, nystagmus, astigmatism	N	Alternating divergent strabismus, significant astigmatism, R amblyopia	N	Cerebral visual defect with erratic eye movements (alternating hyperopia)	3: Impaired vision, strabismus
Fundus examination	Normal	NA	Pale papillae, very thin retina	NA	Normal	NA
VEP/ERG	Immature/normal	NA	Immature, asymmetric/abnormal on the left side	NA	NA	NA

*Neuro/development*						
Delayed development	Y	Y	Y	Y	Y	9/9
Hypotonia	Y (in infancy)	NA	Y	N	Y	6/9
Global motor problems	Walking not acquired	NA	Y	Motor delay	Inability to walk without support	9/9
Fine motor problems	Y	NA	Y	N	Mild difficulties	9/9
Language abilities	No language	Able to do simple sentences	No language	Bisyllabic words at 29 months	Only a few words	Impaired in 9/9
Regression	N	Y (regression of language after callosotomy)	Y (loss ability to walk at age 6 y)	N	N	N
ID degree	Severe	Severe	Severe	NA	Severe	2: Mild; 7: severe
Behavioral problems/ASD features/ADHD	Y (stereotypic movements)	Y (ritualized, rigid nature)	Y (auto- and heteroaggressiveness, stereotypies)	N	Y	1: ASD; 7 ADHD; 1: Attention deficit; 2: Aggressive behavior
Social interactions	Good	Good	Poor contact	Normal	Impaired	Impaired in 2/9
Abnormal movements	N	NA	N	N	N	N
Ataxia	N	NA	Y (ataxic gait)	N	N	N
Pyramidal signs	Y (spasticity, brisk tendon reflexes)	NA	Y (spasticity, brisk tendon reflexes)	N	N	N
Epilepsy/age at onset/type/treatments	11 years/GTCS/sodium valproate levetiracetam	1 year/refractory seizures/LGS/callosotomy at age 18 years, currently lacosamide, pregabalin, carbamazepine, brivaracetam + VNS	4 years/GTCS/focal seizures/sodium valproate	N	7.5 years/tonic upward gaze and disconnection of the environment/lamotrigine	1: Epilepsy at 3 years/sodium valproate
EEG	Interictal slow wave activity	Slow background activity, multifocal interictal epileptic discharges, video EEG consistent with LGS	Slow wave activity, spikes	—	Slow background activity, focal paroxysmal spike–waves, and polyspike–waves in frontocentral regions tending to diffusion during sleep	1: Epileptiform activity
Brain MRI (at age)	Cortical atrophy (3.5 years)	Sequela of callosotomy mild asymmetry of hippocampi (35 years)	Partial agenesis of corpus callosum, global cortical atrophy, myelination delay, epiphyseal cyst, pars intermedia cyst (< 1 year)	Mild nonspecific bilateral flair hypersignal of the posterior parietal periventricular white matter (27 months)	Normal (12 years)	Mild, nonspecific anomalies in 4/6 (delayed myelination, mild hypoplasia of corpus callosum and vermis, abnormal signals in the cortices)

Abbreviations: ADHD: attention deficit hyperactivity disorder; ASD: autism spectrum disorder; EEG: electroencephalogram; ERG: electroretinogram; F: female; GTCS: generalized tonic–clonic seizures; ID: intellectual disability; LGS: Lennox-Gastaut syndrome; M: male; MRI: magnetic resonance imaging; N: no; NA: not available or not applicable; SD: standard deviation; VEP: visual evoked potentials; VNS: vagal nerve stimulation; Y: yes.; y: years.

## Data Availability

The authors confirm that the data supporting the findings of this study are available within this article and its supporting information or available upon request without undue reservation except for exome sequencing data due to privacy and ethical restrictions.

## General Statement

*Web Resources*. The web resources are as follows: GeneMatcher, https://genematcher.org/; gnomAD Browser v2.1.1, https://gnomad.broadinstitute.org/; LOVD (Leiden Open Variation database) https://www.lovd.nl/; and MobiDetails, https://mobidetails.iurc.montp.inserm.fr/MD/.

## Supporting Information

Supporting Information Additional supporting information can be found online in the Supporting Information section. Supplementary Table 1: Oligonucleotide sequences used to generate *ATP9A* missense variants by site-directed mutagenesis. Supplementary Table 2: In silico prediction of pathogenic effect of *ATP9A* missense mutations. Supplementary Figure 1: Sanger sequencing and segregation analysis of the missense variants showing their de novo occurrence. Supplementary Figure 2: Subcellular localization of WT or mutant forms of ATP9A protein overexpressed in neuronal cells. 3D images were obtained using a Leica confocal microscope (objectives × 63, scale bar = 10*  μ*m). Clinical description of the patients.
