# Supplementary material for: Heterozygous Missense Variants in the ATPase Phospholipid Transporting 9A Gene, ATP9A, Alter Dendritic Spine Maturation and Cause Dominantly Inherited Nonsyndromic Intellectual Disability
Source: Hum Mutat. 2025 Mar 5;2025:7085599. doi: 10.1155/humu/7085599 (PMC11987072; doi:10.1155/humu/7085599)
Supplement: Supporting Information — Additional supporting information can be found online in the Supporting Information section. Supplementary Table 1: Oligonucleotide sequences used to generate ATP9A missense variants by site-directed mutagenesis. Supplementary Table 2: In silico prediction of pathogenic effect of ATP9A missense mutations. Supplementary Figure 1: Sanger sequencing and segregation analysis of the missense variants showing their de novo occurrence. Supplementary Figure 2: Subcellular localization of WT or mutant forms of ATP9A protein overexpressed in neuronal cells. 3D images were obtained using a Leica confocal microscope (objectives × 63, scale bar = 10 μm). Clinical description of the patients. [file 7085599.f1.docx]

**Supplementary data**

**Heterozygous missense variants in the ATPase phospholipid transporting 9A gene, *ATP9A*, alter dendritic spine maturation and cause dominantly inherited non syndromic intellectual disability**

Amélie Cordovado, Yvan Hérenger, Coline Cormier, Estrella López-Martín, Hannah Stamberger, Laurence Faivre, Anne-Sophie Denommé Pichon, Antonio Vitobello, Hamza Hadj Abdallah, Giulia Barcia, Thomas Courtin, Beatriz Martínez-Delgado, Eva Bermejo-Sánchez, María J. Barrero, Brooklynn Gasser, Stéphane Bezieau, Sébastien Küry, Sarah Weckhuysen, Frédéric Laumonnier, Annick Toutain, Marie-Laure Vuillaume.

Supplementary Table S1: Oligonucleotide sequences used to generate ATP9A missense variants by site-directed mutagenesis

| Variant |  | Primer F | Primer R |  |  |
| --- | --- | --- | --- | --- | --- |
| c.1178C>G | p.(Trp393Arg) | 5’-ACAGACAAGAgAGGCACTCTTAC | 5’-GAGTAAGTACGAAATCCTGC |  |  |
| c.1198G>C | p.(Glu400Gln) | 5’-TACCCAGAACcAGATGATTTTCAAAC | 5’-AGAGTGCCTGTCTTGTCTG |  |  |
| c.1381A>G | p.(Lys461Glu) | 5’-CGAAGCCGTGgAGGCCATCGC | 5’-TGCACGCGGCTGCTCATG |  |  |
| c.1655G>C | p.(Gly552Ala) | 5’-AAACGTATGGcCATCATCGTG | 5’-GCTTTCATAGGTGAAAGG |  |  |
|  |  |  |  |  |  |

Supplementary Table S2: In silico prediction of pathogenic effect of ATP9A missense mutations. Numbering is according to the cDNA sequence (GenBank entry NM_006045.3).

| **Position** | **Nucleotide  change** | **Predicted amino  acid change** | **GnomAD  v2.1.1** | **SIFT v6.2.0** | **Mutation Taster v2021** | **Polyphen 2 (HumVar)** | **Revel** | **CADD score** |
| --- | --- | --- | --- | --- | --- | --- | --- | --- |
| exon 12 | c.1178C>G | p.(Thr393Arg) | Absent | Damaging (0) | Deleterious 78/22 (del/benign) | Probably damaging (0.995) | Damaging (0,903) | 32 |
| exon 13 | c.1198G>C | p.(Glu400Gln) | Absent | Damaging (0.004) | Deleterious Tree vote 57/43 (del/benign) | Probably damaging (0.929) | Uncertain (0.273) | 26.70 |
| exon 14 | c.1381A>G | p.(Lys461Glu) | Absent | Tolerated (0.617) | Deleterious Tree vote 55/45 (del/benign) | Benign (0.050) | Uncertain (0.373) | 22.80 |
| exon 15 | c.1655G>C | p.(Gly552Ala) | Absent | Damaging (0.008) | Deleterious Tree vote 86/14 (del/benign) | Probably damaging (0.999) | Damaging (0.711) | 26.80 |
| exon 20 | c.2137C>G | p.(His713Asp) | Absent | Tolerated (0.053) | Deleterious Tree vote 70/30 (del/benign) | Probably damaging (0.959) | Damaging (0.647) | 28.4 |
|  |  |  |  |  |  |  |  |  |

Source: Mutation Taster v2021 (Alamut Visual +), SIFT, Polyphen 2, Revel and CADD score (MobiDetails)


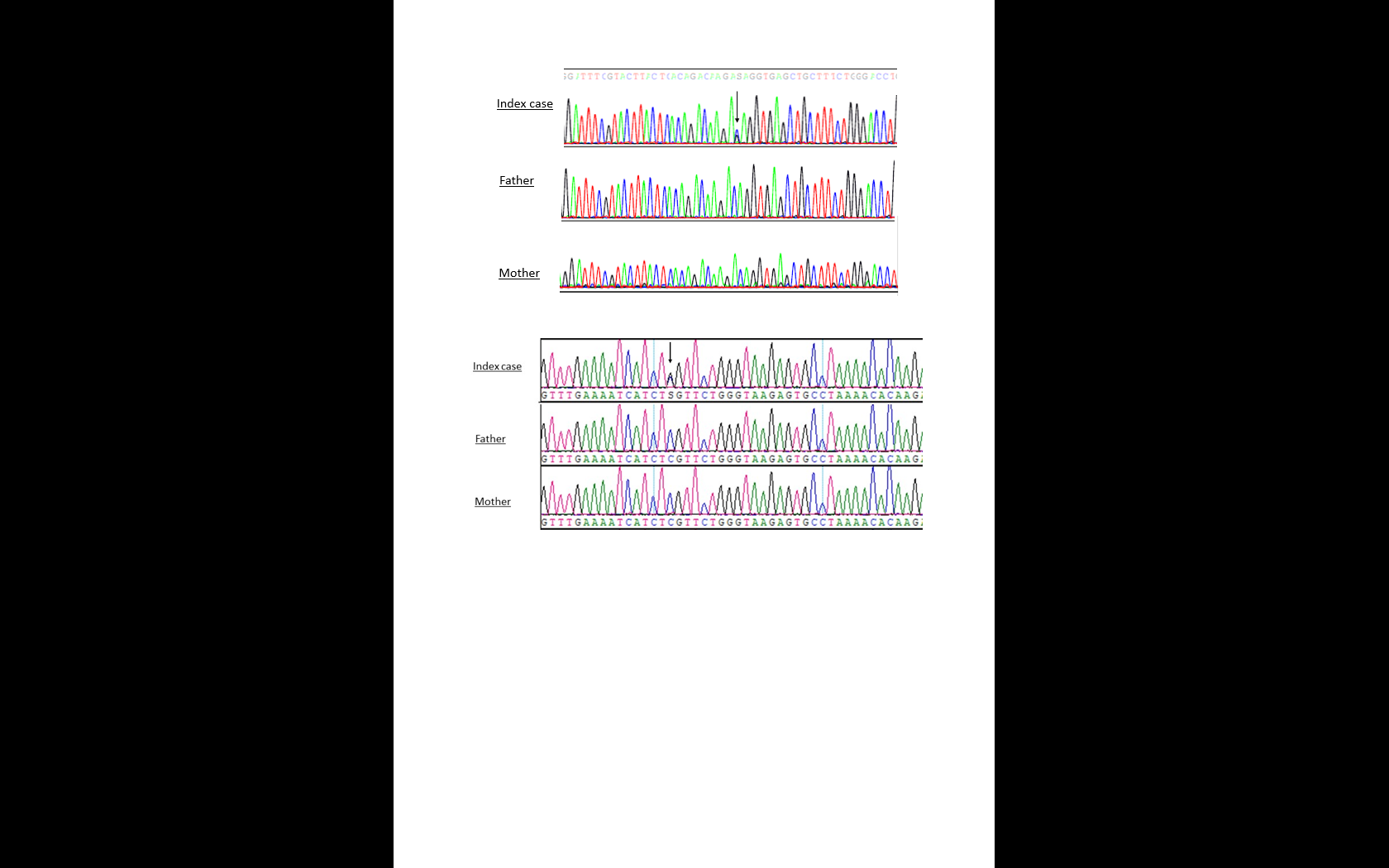

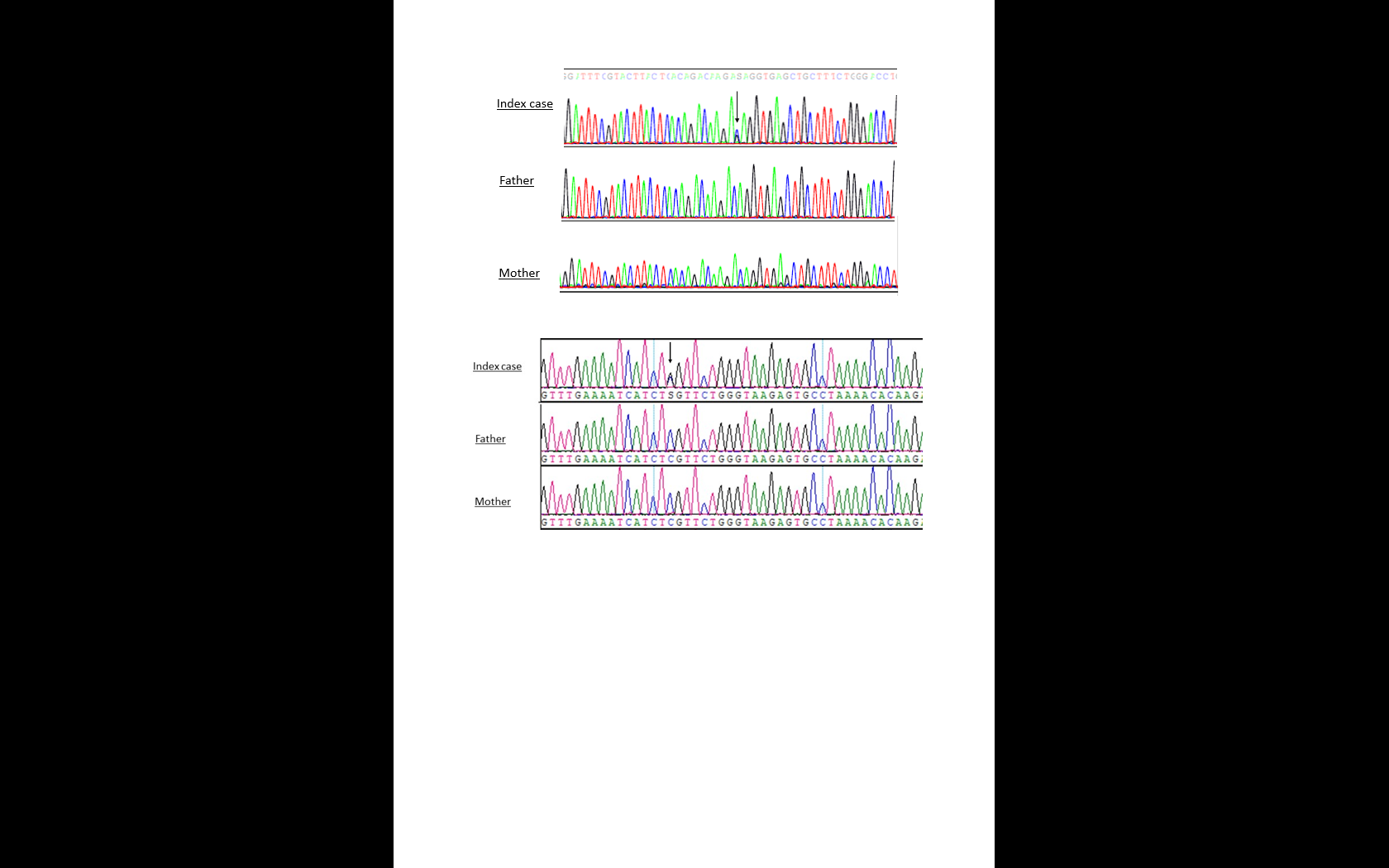

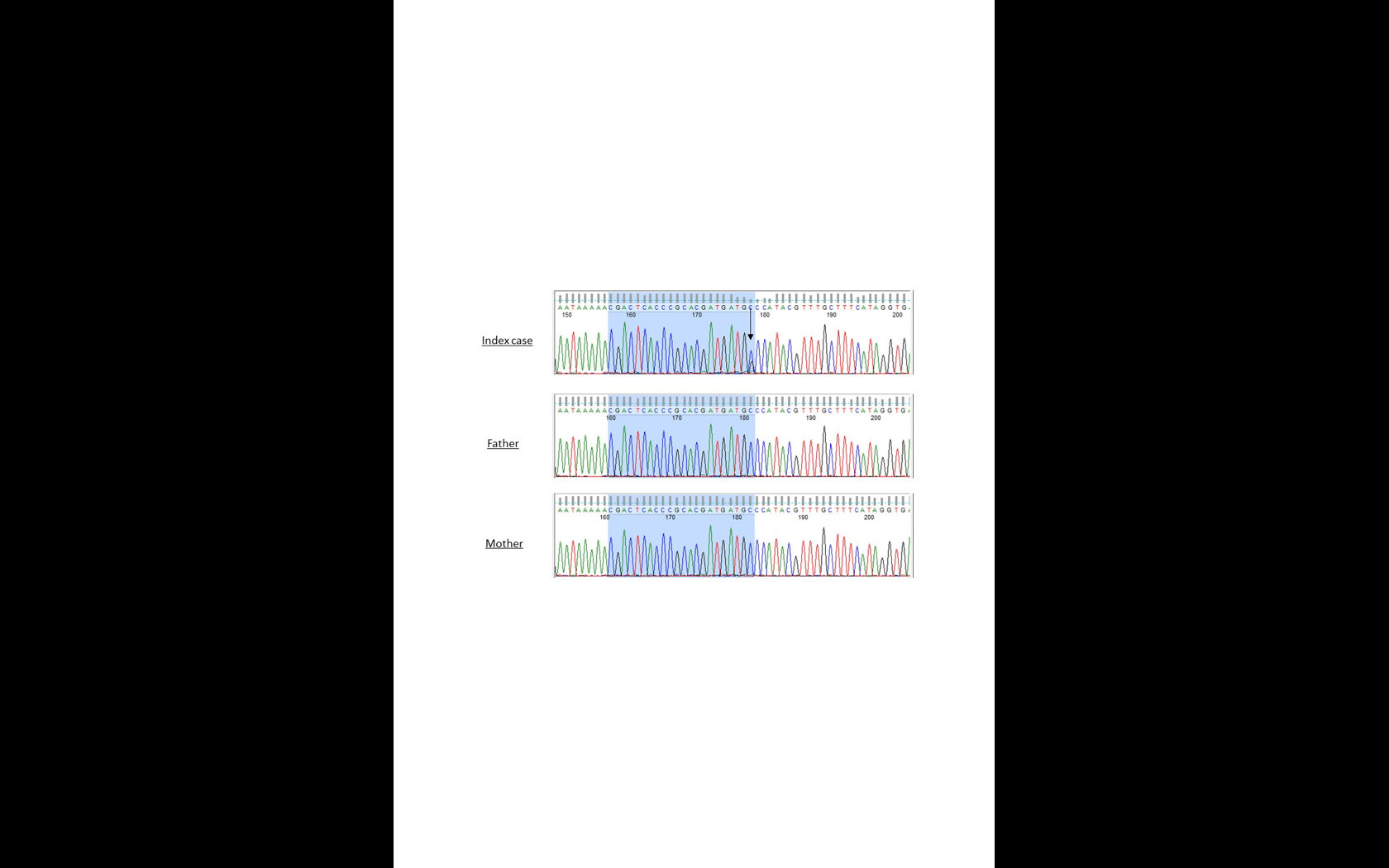

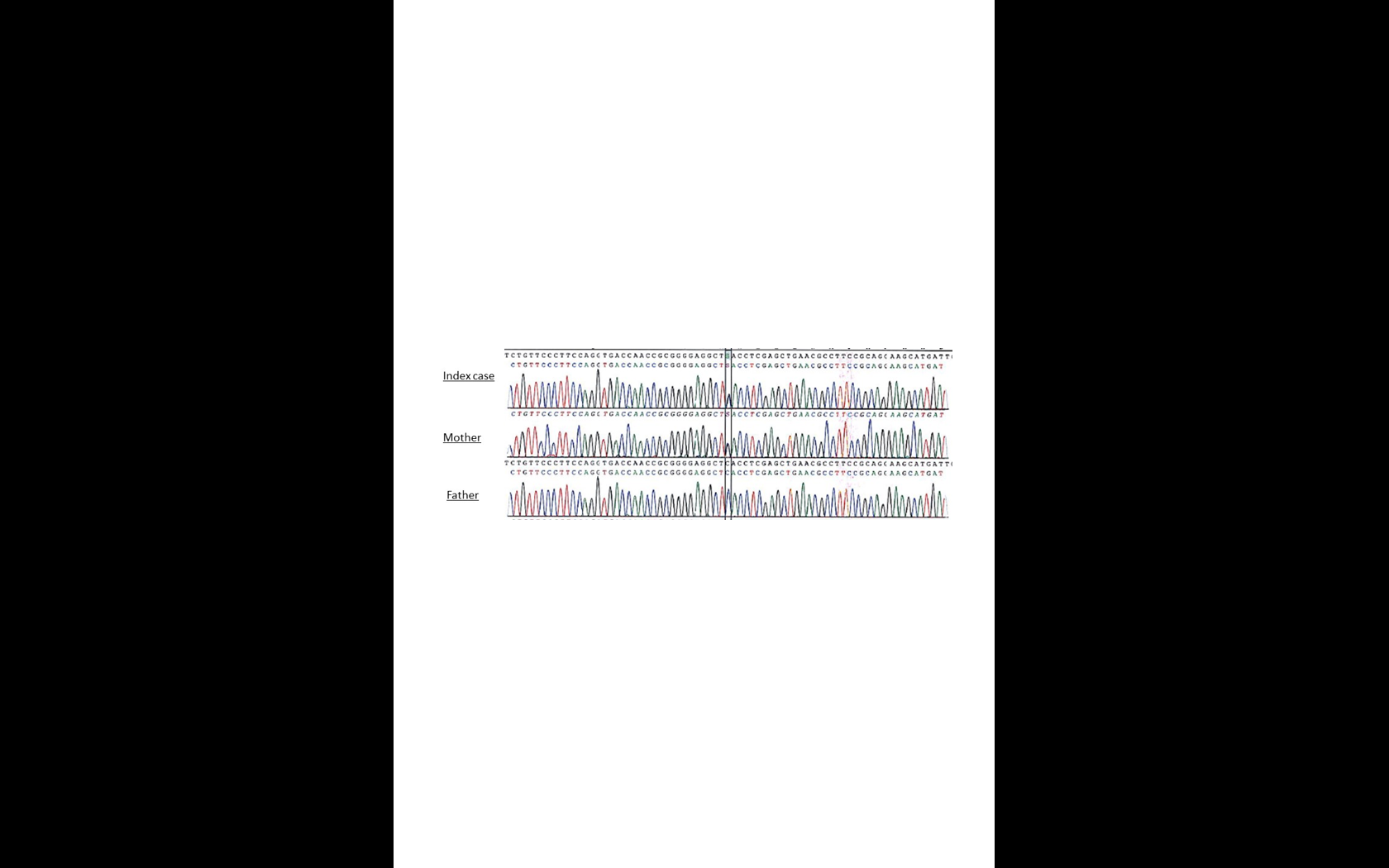


**Variant NM_006045.3 : c.1178C>G, p.(Thr393Arg)**

**Variant NM_006045.3 : c.1198G>C, p.(Glu400Gln)**

**Variant NM_006045.3 : c.1655G>C, p.(Gly552Ala)**

**Variant NM_006045.3 : c.2137C>G, p.(His713Asp)**

**Supplementary Figure 1:** Sanger sequencing and segregation analysis of the missense variants showing their de novo occurrence

**
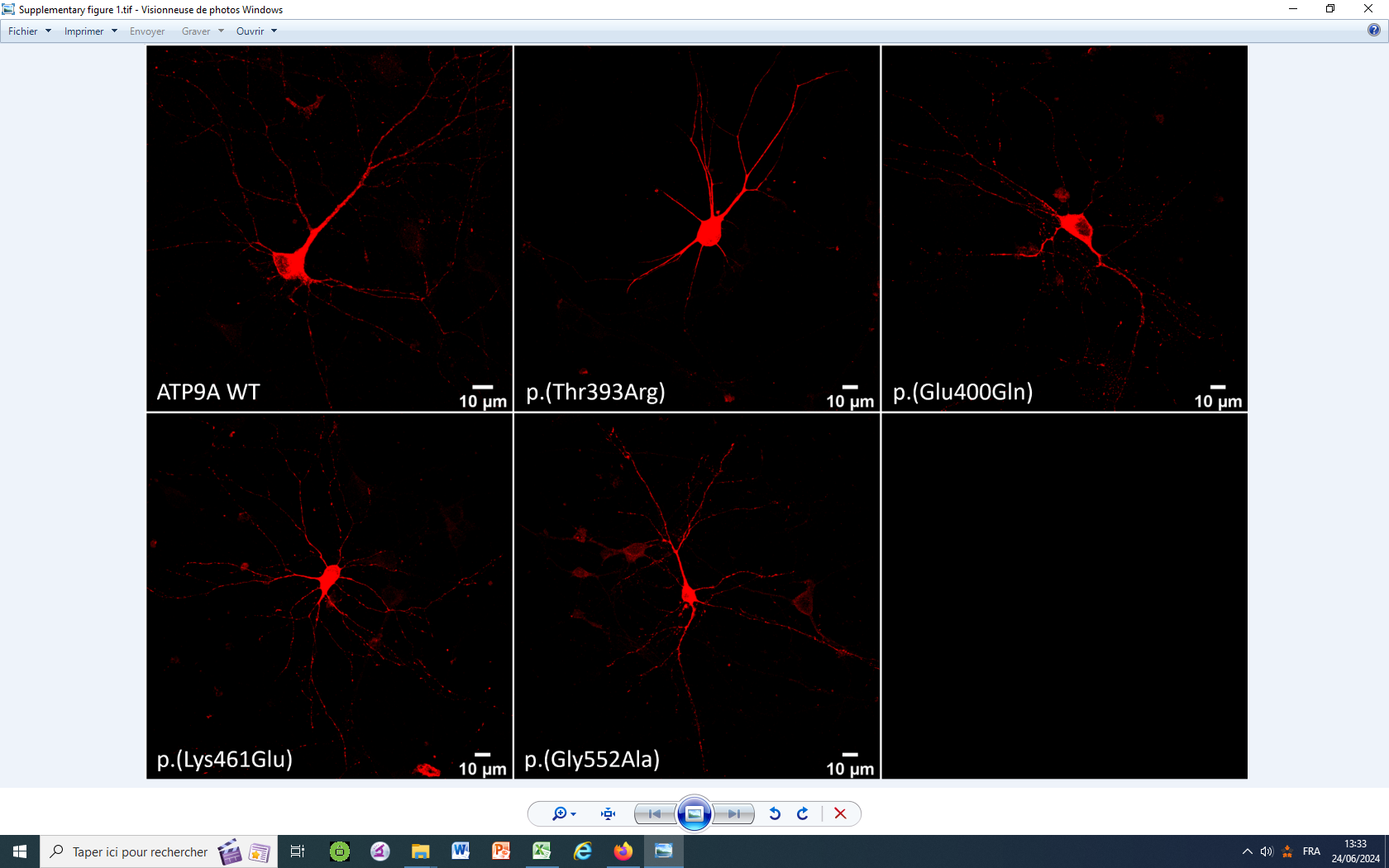
**

**Supplementary Figure 2:** Subcellular localization of wild type or mutant forms of ATP9A protein overexpressed in neuronal cells. 3D-Images were obtained using a Leica Confocal microscope (objectives x63, scale bar = 10 µm).

**Clinical descriptions**

**Patient 1**

Male patient aged 16 years. Second child of healthy unrelated Caucasian parents with no family history. An elder sister and a younger brother have normal development.

Normal pregnancy. Delivery at 38 weeks by caesarean section. Apgar score at 10. Normal neonatal examination. Birth measurements in the low normal values: BL = 46 cm (5^th^-10^th^ percentile), BW = 2770 g (10^th^-25^th^ percentile), BOFC = 32 cm (5^th^ percentile).

Medical problems since birth

- bronchiolitis at 9-10 months; allergic rhinitis (as his mother and sister),

- severe feeding difficulties with gastro-oesophageal reflux and food aversion responsible for weight loss, and requiring tube feeding and then gastrostomy at the age of 4 years,

- then statural growth at – 2 SD i.e. one SD below expected height; weight at + 1 SD/height,

- teeth malposition due to lack of place (small mandibule),

- severe right thoracolumbar scoliosis requiring arthrodesis at the age of 13 ½ years,

- progressive joint retractions (knees, ankles, elbows) and reducible hyperextension of the wrists (more severe on the left).

Neurological problems/Development

- global delay and hypotonia noticed at the age of 9-10 months. Sitting acquired at the age of 14 months. At the age of 16 years is unable to walk, not able to stand up without support.

- no language

- not toilet-trained

- stereotypic movements: flapping of hands, taps his ears or his mouth

- smiles, laughs, has good interactions

- microcephaly around – 3 SD

- progressive spasticity of all 4 limbs responsible for joint retractions

- epilepsy consisting of generalized tonic-clonic seizures since the age of 11 years, treated with sodium valproate and levetiracetam

- special education.

Vision

- convergent strabismus diagnosed at 6-8 months, treated by glasses and orthoptic rehabilitation; astigmatism; multidirectional nystagmus,

- visual acuity impossible to evaluate

Physical examination

- normal heart sounds, abdominal palpation, skin examination, genitalia,

- no particular dysmorphic craniofacial features: normal skull, oval shape face, rather large ears but normally shaped, slightly almond-shaped palpebral fissures, no abnormality in the oral cavity except for teeth malposition,

- normal limbs and extremities except for joint retractions developing with age.

Investigations

- brain MRI at the age of 3 years: global cortical atrophy (ventricular dilatation and large Sylvian fissures), normal corpus callosum, no delay in myelination,

- EEG: interictal slow background activity,

- normal fundus examination and ERG; immature VEP,

- normal hearing tests,

- normal heart and abdominal ultrasound scan,

- skeletal X-rays: normal skull, coxa valga, scoliosis,

- normal routine blood tests,

- normal lactate & pyruvate, ammonia, thyroid hormones, GH, blood and urinary amino acid chromatography, urinary organic acid chromatography, urinary creatine & guanidinoacetate, CK, transferrin isoforms, VLFA, SAICAR, MPS.

Genetic analyses

- normal chromosome analysis, *FMR1* analysis, MLPA of *MECP2*, 15q11 region methylation,

- array-CGH: maternally inherited 185 kb 9q22.32 duplication,

- trio exome sequencing in a research setting showed a *de novo* heterozygote *ATP9A* variant [chr20(hg19):g.50287656G>C; NM_006045.3: c.1178C>G; NP_006036.1:p.(Thr393Arg)].

**Patient 2**

Female patient, now 49 years old. Third child of healthy non-consanguineous Caucasian parents. No relevant family history. Two older sisters had normal development and no epilepsy.

Normal pregnancy. Delivery at term with caesarean section due to face forward position. Unclear if there were problems at birth (as reported by the parents, she initially had a blue color, but she did not require prolonged hospitalization).

Medical problems

- Gall stones

- Overweight/obesity

Neurological problems/development

Seizures started at around 1 year of age, were difficult to control with anti-epileptic medications and led to several hospitalizations over the years. Over time, the patient had multiple seizures a day including myoclonic and tonic-clonic seizures and drop attacks for which she needed a helm. A diagnosis of Lennox Gastaut syndrome was made at the age of 17-18 years old. Following a severe status epilepticus at the age of 18 years, she underwent a partial callosotomy without a lasting effect. When she was 24 years old a vagal nerve stimulator (VNS) was implanted which had a partial effect on seizure frequency, and perhaps more significantly increased alertness. Atkins diet, when she was 32 years old, was not tolerated and was stopped after several weeks. She currently still has daily myoclonic seizures and smaller focal seizures and weekly (nocturnal) (focal to) bilateral tonic-clonic seizures. She is currently treated with the combination of brivaracetam, pregabaline, carbamazepine, and lacosamide.

The patient had normal development during the first year of life but her development slowed down after the onset of seizures. She had periods of regression, including loss of language skills, after her callosotomy or after medication changes, but improvement when certain medications were discontinued. VNS also had a very good effect on her overall alertness and on her speech and language. She had severe intellectual disability. She could walk independently indoors but used a wheelchair for longer distances. She was able to do short sentences. She was able to eat and drink independently and to go to the toilet with help, and weared incontinence material. She was said to be a social person but with a rather rigid nature requiring a fixed schedule. She had periods with behavioral problems, associated with recurrence of (difficult to control) seizures.

Physical examination showed no obvious dysmorphic features.

Investigations

- brain MRI at 35 years: loss of tissue on the right frontal lobe related to the callosotomy. Hemosiderin deposition in the temporal horn and the anterior wall of the right ventricle. Slight asymmetry of hippocampi (right slighly smaller).

- EEG: prior EEGs showed moderately to severely slow background activity. Several EEGs showed multifocal epileptic discharges.

- 48-hour EEG monitoring at the age of 41 years: multiple brief seizures during which myoclonic or tonic tensing of a limb could be seen.

- Polysomnography at 41 years: awakening reactions partially triggered by hypopnea, partially by seizures and in part with no obvious cause. Manifest ronchopathy especially on supine position.

Genetic analyses in a research setting:

- *SCN1A*, *PCDH19*, *STXBP1* and epilepsy gene panel screening: negative

- SNP array: heterozygote *PLCB1* variant (gene associated with an autosomal recessive condition)

- trio exome sequencing showed a *de novo* *ATP9A* variant [Chr20(hg19):g.50286631C>G; NM_006045.3:c.1198G>C; NP_006036.1:p.(Glu400Gln)].

**Patient 3**

Female patient aged 15 years. Second child of healthy twice-related North-African parents. An older sister, a younger sister and a younger brother have normal development. A paternal cousin has developmental delay and severe hydrocephalus, detected at 22 weeks of gestation, caused by aqueduct of Sylvius stenosis which required a ventriculoperitoneal shunt.

Normal pregnancy. Delivery at 38 weeks of gestation. APGAR score at 9 and 10. Normal neonatal examination. Birth measurements in the high normal values: BL = 51cm (91^th^ percentile – AUDIPOG), BW = 3640g (91 ^th^ percentile – AUDIPOG), BOFC = 35cm (82 ^th^ percentile).

Neurological problems/Development

- normal development until the age of 6 months. Global delay and hypotonia noticed at the age of 9 months. Sitting acquired at the age of 11 months. Walking acquired after the age of 2 years then followed by regression with loss of walking at 6 years.

- no language

- stereotypic movements around the median line, anterior and posterior rocking behavior, teeth chattering without bruxism

- poor interactions

- progressive spasticity requiring botulinum toxin injections and tenotomies

- epilepsy consisting of generalized and partial tonic-clonic seizures since the age of 4 years, treated with sodium valproate.

- special education

Vision

- alternating divergent strabismus; significant astigmatism; amblyopia in right eye

- pale papillae and very thin retina on fundus examination

Physical examination

- normal heart sounds, abdominal palpation, skin examination,

- hyperlaxity of both wrists

- minor dysmorphic craniofacial features: anteverted nares and preauricular fistula

- normal limbs and extremities except for joint retractions developing with age,

- brisk tendon reflexes, Babinski sign on the left side.

Investigations

- brain MRI before the age of 1 year: partial agenesis of the corpus callosum, global cortical atrophy, myelination delay, epiphyseal cyst, pars intermedia cyst

- EEG: slow background activity overloaded with slow-wave peaks

- auditory evoked potentials at 11 years suggesting left auditory neuropathy with thresholds around 60 dB bilaterally

- visual evoked potentials: immature morphology, asymmetry at the expense of the right occipital derivation, consistent with agenesis of the corpus callosum

- ERG: abnormal on left side

- normal routine blood tests

- metabolic tests: plasma amino acid analysis showed hyperaminoacidemia, normal urinary organic acid chromatography, normal lactate.

Genetic analyses

- normal chromosome analysis

- array-CGH: benign CNV (184kb 9p21.2p21.1 duplication)

- trio exome sequencing in a diagnostic setting: *de novo* heterozygote *ATP9A* variant [chr20(hg19):g.50255895C>G; NM_006045.3:c.1655G>C; NP_006036.1:p.(Gly552Ala)].

**Patient 4**

Male patient, 29 months old. Second child of unrelated French Caucasian parents with no family history. His older sister has a normal development.

The mother had gestational diabetes during pregnancy. Normal delivery at 39 weeks of gestation. Apgar scores at 10-10. Normal neonatal examination. Birth measurements: BL = 50 cm (50^th^ percentile), BW = 4100 g (95^th^ percentile), OFC = 35 cm (50^th^ percentile).

Neurological problems/Development

- No neonatal history.

- Normal development until the age of 8 months. Global motor delay with sitting acquired at the age of 13 months, and walking acquired after the age of 24 months.

- Language limited to bisyllabic words and no sentences at the age of 29 months. Undertands simple orders.

- No regression reported

- Toilet trained at day time but nocturnal enuresia

- Normal social interactions

- No tonus or movement abnormalities

- No history of seizure

- Special education, speech therapy and physiotherapy.

Physical examination

- Height: 93 cm (+1 SD), Weight: 18.6 kg (+ 4.8 SD/height) and HC: 50 cm (+ 0.4 SD)

- normal heart sounds, abdominal palpation, skin, limbs and neurological examination.

- no dysmorphic craniofacial features

- sacro-coccygeal dimple.

Investigations

- brain MRI before the age of 27 months: minimal and non-specific bilateral FLAIR hyperintensities of the posterior parietal periventricular white matter,

- normal spinal cord MRI,

- normal auditory evoked potentials

Genetic analyses

- normal array-CGH (60 Kb resolution)

- FRAXA excluded

- whole genome sequencing: *de novo* heterozygote *ATP9A* variant [ chr20(hg19):g.50235561G>C; NM_006045.3: c.2137C>G; NP_006036.1:p.(His713Asp)]

**Patient 5**

The patient is a 12-year-old female, only child of healthy non-consanguineous Caucasian parents with no family history.

She was born at term after a controlled pregnancy due to a maternal urinary tract infection detected during the third trimester. Apgar score 9/10. Birth measurements were: weight, 2510 g (p2, -2.07 SD), length, 45 cm (p‹1, -2.9 SD), and head circumference, 34 cm (p36, -0.37 SD). At 48 hours of age, she had multifactorial jaundice, mild dehydration, and mild hypernatremia.

Medical problems

- Severe feeding difficulties with gastro-esophageal reflux, dysphagia and episodic vomiting. Chronic diarrhea.

- Anemia detected since birth by systematic blood investigations might result from her diarrhea episodes and severe feeding difficulties. This problem is constant, although her serum iron concentration increases when she is treated with oral iron supplementation.

- Hypogammaglobulinemia, constant from birth until he was 5-6 years old. From this age, this problem has been transient. She has never been treated for hypogammaglobulinemia.

- Failure to thrive diagnosed at 2 months of age. On last examination, at 10 years, height was at – 2.67 SD (p‹1) and weight at -2.05 SD (p3).

Neurological problems/Development

Psychomotor delay noticed at the age of 2 months. Head control was achieved at 3 months and sitting acquired at the age of 10 months. She was able to walk at the age of 43 months. Currently, she is unable to walk without support and she has mild difficulties in fine motor skills. She could speak single words for the first time at 4 years, although she has never been able to made a sentence. She is not toilet-trained. When she was 7.5 years old, she had her first epileptic crises and, since then, these episodes have been repeated approximately once a month. No anomalies were detected in subsequent electroencephalograms carried out while the child was awake. However, an electroencephalogram during sleep showed paroxysmal spike-wave activity in the frontocentral region. She has diurnal crises consisting in tonic upward gaze and disconnection of the environment, which are treated with lamotrigine. She has abnormal behavior and social interactions, and hyperactivity. She needs special education.

Vision

- erratic eye movements due to a cerebral visual defect

- alternating hyperopia).

Physical examination: no particular dysmorphic craniofacial features, normal limbs. Cephalic perimeter measured at 5 years of age was 48 cm (- 1.9 SD).

Investigations

- Brain MRI at the age of 12 years: normal.

- EEG: Moderate slowing of the basal brain bioelectrical activity. Paroxysmal focal spike-waves and polyspike-waves in the bilateral frontocentral region tending to diffusion, observed during sleep.

- normal fundus examination.

- Blood tests: anemia and hypogammaglobulinemia.

Genetic analyses

- normal chromosome analysis.

- normal 60K and 180K array-CGHs.

- testing for Angelman syndrome, *SHOX* gene, Prader-Willi syndrome and fragile X syndrome yielded normal results.

- clinical exome sequencing in the proband revealed 2 pathogenic *ATP9A* variants, one occurring *de novo* [chr20(hg19):g. 50329508G>A; NM_006045.3:c.433C>T; NP_006036.1:p.(Arg145*) and one inherited from one parent [chr20(hg19):g. 50225101C>A; NM_006045.3:c.2701G>T; NP_006036.1:p.(Glu901*)]. In addition exome sequencing showed 2 pathogenic heterozygous variants in the *PMM2* gene (associated with an autosomal recessive disorder), but both were maternally inherited.
